# 787. *Next Steps:* Teaching Future Generations an Interdisciplinary Approach to Diabetic Foot Ulcer Care

**DOI:** 10.1093/ofid/ofad500.848

**Published:** 2023-11-27

**Authors:** Shalvi B Parikh, Jamie N LaMantia, Meghan B Brennan, Jessica S Tischendorf

**Affiliations:** University of Wisconsin-Madison School of Medicine and Public Health, Madison, Wisconsin; University of Wisconsin-Madison School of Medicine and Public Health, Madison, Wisconsin; University of Wisconsin, Department of Medicine, Madison, Wisconsin; University of Wisconsin School of Medicine and Public Health, Madision, Wisconsin

## Abstract

**Background:**

Interdisciplinary care of diabetic foot ulcers (DFUs) is associated with nearly 40% reduction in major (above ankle) amputations. We developed a curriculum emphasizing interprofessional collaboration (IPC) between Infectious Disease (ID) fellows and Podiatry residents in the care of veterans with DFUs.

**Methods:**

The curriculum was delivered in two one-hour in-person sessions. The first session provided background on principles of IPC and perspective sharing of other professions through case discussions. The second session, co-facilitated by an ID physician and a podiatrist, allowed ID fellows and podiatry residents to work through DFU cases. We used a quasi-experimental design (Figure 1) and a multipronged assessment designed by applying Miller's pyramid (Figure 2). We used a modified Jefferson Scale of Attitudes toward IPC (Likert scale 1-5, 5 = strongly positive) to measure fellow attitudes, with items subcategorized by themes of shared learning and diversity & ethics. Fellows reported on the frequency of communication with the primary and podiatry team weekly, and charts of veterans with DFUs were reviewed for documentation of ID-specific content (organism and antibiotic plan), as well as non-ID specific plans (glycemic control, vascular status, and biomechanical considerations). Two-sided t-test was used to compare pre-post performance.

**Figure:**
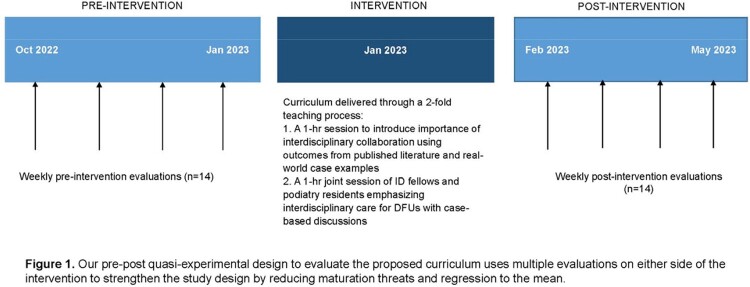

**Figure:**
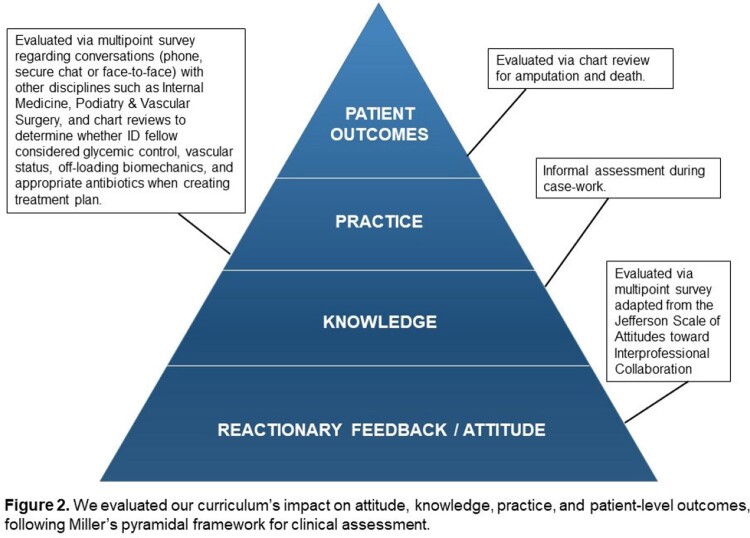

**Results:**

Five ID fellows participated. Post-session evaluations suggest the curriculum was viewed favorably. Fellows had strongly positive baseline attitudes toward IPC. Attitudes toward shared learning improved (4.13 vs 4.44, p < 0.01), but diversity & ethics did not (4.86 vs 4.82, p = 0.61). Hospitalizations of seven veterans with DFUs were analyzed pre-intervention and four analyzed post-intervention. ID-specific content and communication with the primary team remained 100% over the study period. Non-ID-specific content documentation remained low (20% vs 11%, p = 0.30). Frequency of communication with podiatry did not change significantly (57% vs 25%, p = 0.35).

**Conclusion:**

Interdisciplinary didactic sessions were well-received and may improve attitudes toward shared learning, but likely need to be augmented with interprofessional clinical experiences to achieve practice improvements in the care of veterans with DFUs.

**Disclosures:**

**All Authors**: No reported disclosures

